# Hydrogels in Cardiac Surgery: Versatile Platforms for Tissue Repair, Adhesion Prevention, and Localized Therapeutics

**DOI:** 10.3390/gels11070564

**Published:** 2025-07-21

**Authors:** Seok Beom Hong, Jin-Oh Jeong, Hoon Choi

**Affiliations:** 1 Department of Thoracic and Cardiovascular Surgery, Seoul St. Mary’s Hospital, College of Medicine, The Catholic University of Korea, Seoul 06591, Republic of Korea; seok_beom@naver.com; 2 Wake Forest Institute for Regenerative Medicine (WFIRM), Wake Forest School of Medicine, Winston-Salem, NC 27157, USA; jijeong@wakehealth.edu; 3 Department of Anesthesiology and Pain Medicine, Seoul St. Mary’s Hospital, College of Medicine, The Catholic University of Korea, Seoul 06591, Republic of Korea

**Keywords:** biomaterials, tissue engineering, cardiac regeneration, valve scaffold, pericardial barrier, controlled release, extracellular matrix

## Abstract

Hydrogels have emerged as multifunctional biomaterials in cardiac surgery, offering promising solutions for myocardial regeneration, adhesion prevention, valve engineering, and localized drug and gene delivery. Their high water content, biocompatibility, and mechanical tunability enable close emulation of the cardiac extracellular matrix, supporting cellular viability and integration under dynamic physiological conditions. In myocardial repair, injectable and patch-forming hydrogels have been shown to be effective in reducing infarct size, promoting angiogenesis, and preserving contractile function. Hydrogel coatings and films have been designed as adhesion barriers to minimize pericardial adhesions after cardiotomy and improve reoperative safety. In heart valve and patch engineering, hydrogels contribute to scaffold design by providing bio-instructive, mechanically resilient, and printable matrices that are compatible with 3D fabrication. Furthermore, hydrogels serve as localized delivery platforms for small molecules, proteins, and nucleic acids, enabling sustained or stimuli-responsive release while minimizing systemic toxicity. Despite these advances, challenges such as mechanical durability, immune compatibility, and translational scalability persist. Ongoing innovations in smart polymer chemistry, hybrid composite design, and patient-specific manufacturing are addressing these limitations. This review aims to provide an integrated perspective on the application of hydrogels in cardiac surgery. The relevant literature was identified through a narrative search of PubMed, Scopus, Web of Science, Embase, and Google Scholar. Taken together, hydrogels offer a uniquely versatile and clinically translatable platform for addressing the multifaceted challenges of cardiac surgery. Hydrogels are poised to redefine clinical strategies in cardiac surgery by enabling tailored, bioresponsive, and functionally integrated therapies.

## 1. Introduction

Hydrogels are a versatile and rapidly advancing class of biomaterials that offer distinct advantages for tissue repair and regeneration in diverse clinical fields. As three-dimensional (3D) hydrophilic polymer networks with a high water content, they closely mimic the extracellular matrix (ECM), enabling biocompatible interactions with cells and tissues [1,2]. Whether derived from natural sources, such as collagen or hyaluronic acid, or synthesized from polymers, such as polyethylene glycol (PEG) and polyvinyl alcohol (PVA), hydrogels exhibit tunable physical and biochemical properties [3,4]. Functionalization with peptides, growth factors, or other bioactive molecules further augments their biological performance [5].

One of their most promising attributes is their potential for minimally invasive delivery. Injectable hydrogels can undergo in situ gelation in response to environmental triggers, such as temperature, pH, or enzymatic activity, allowing for localized and conformable applications at complex anatomical sites [6,7]. Recent innovations in microfabrication and 3D bioprinting have enabled the precise control of hydrogel architecture, porosity, and spatial composition, broadening their therapeutic scope [8,9].

While hydrogels were initially explored for musculoskeletal and dermatological applications, their use in cardiovascular medicine, particularly cardiac surgery, has expanded considerably in recent years. This transition has been driven by unmet clinical challenges, such as myocardial ischemia, poor tissue integration, adverse ventricular remodeling, and postoperative adhesions [10,11]. These persistent complications are further compounded by the limited regenerative capacity of the adult myocardium, necessitating the use of biomaterials capable of providing mechanical support and promoting endogenous repair [12].

In this context, hydrogels have been utilized for a range of functions. They serve as scaffolds for myocardial patches, carriers for stem cells or pharmacological agents, and barriers to reduce pericardial adhesions [13,14]. Some hydrogels have been engineered to match the viscoelastic properties of the native myocardium, thereby facilitating mechanical integration [11]. Others have incorporated ECM-mimetic sequences or conductive components to promote cellular organization and synchronized contraction [15,16]. Emerging bioresponsive hydrogels can dynamically respond to pathophysiological cues, such as oxidative stress or protease activity, enabling spatiotemporally controlled therapeutic delivery [14,17].

Despite this promise, several translational challenges remain. These include mechanical mismatch with the beating heart, insufficient electrical conductivity, immune responses, and challenges related to Good Manufacturing Practice-grade production and long-term biocompatibility [10,18]. Moreover, the regulatory approval of hydrogel-based devices requires extensive validation of sterility, degradation behavior, and safety profiles [19,20].

Nevertheless, growing evidence from large animal models and early-phase clinical studies supports the feasibility and therapeutic potential of hydrogel systems for cardiac applications [10,21]. These developments underscore the critical intersection between biomaterial science, cardiovascular biology, and surgical innovation.

This narrative review aims to provide a comprehensive and up-to-date overview of hydrogel technology in cardiac surgery. We focus on material design strategies, functional outcomes, and translational pathways, organized across the following four thematic domains: (1) clinical challenges and unmet needs, (2) hydrogel types and design features, (3) specific surgical applications, including myocardial repair and pericardial adhesion barriers, and (4) hydrogel-mediated drug and gene delivery. We conclude with key considerations for the clinical translation and future perspectives of this approach.

### Literature Search Strategy

Relevant and recent evidence was extracted from multiple databases, including PubMed, Scopus, Web of Science, Embase, and Google Scholar. The literature search was conducted using combinations of keywords such as “hydrogel”, “cardiac surgery”, “biomaterials”, “myocardial repair”, “adhesion prevention”, “heart valve”, and “drug delivery”. Articles were selected based on their relevance to hydrogel applications in cardiac surgery, encompassing myocardial regeneration, pericardial adhesion barriers, valve engineering, and localized therapeutic delivery. All retrieved studies and relevant review articles were manually screened to identify additional sources of interest. There were no restrictions on article type. Appropriateness for inclusion was determined by the authors to ensure broad, contemporary, and unbiased coverage of the field.

## 2. Cardiac Surgery: Opportunities and Challenges for Hydrogels

### 2.1. Clinical Challenges in Cardiac Surgery

Cardiac surgery remains a cornerstone of cardiovascular medicine, offering definitive treatment for a broad range of structural heart diseases, including coronary artery disease, valvular disorders, congenital defects, and advanced heart failure. Standard procedures, such as coronary artery bypass grafting, valve repair or replacement, septal defect closure, and left ventricular assist device implantation, have significantly improved the survival and quality of life of millions of patients [22]. Despite these successes, modern cardiac surgery still faces major challenges arising from the fragility of myocardial tissue, systemic effects of cardiopulmonary bypass, and limited regenerative capacity of the heart [23,24].

Surgical trauma initiates a highly regulated but frequently maladaptive wound-healing cascade. Ischemia–reperfusion injury, cardioplegic arrest, and mechanical manipulation result in acute inflammation marked by endothelial activation, leukocyte infiltration, and the release of cytokines such as tumor necrosis factor-alpha (TNF-α) and interleukin (IL)-1β [25,26]. This inflammatory response activates fibroblasts and promotes the deposition of ECM proteins, primarily collagen types I and III, which can culminate in pathological fibrosis [27,28]. Myofibroblast activation, driven by transforming growth factor-beta (TGF-β), mechanical stress, and proinflammatory cues, further exacerbates ventricular stiffening, conduction disturbances, and contractile dysfunction [29,30,31].

Postoperative complications such as pericardial adhesions add further complexity. Fibrotic bridging between the epicardium and surrounding structures impairs surgical access and increases the risk of myocardial injury during reoperation [32]. Moreover, synthetic materials commonly used in cardiac surgery, including prosthetic valves, vascular grafts, and patches, often lack bioactivity, leading to poor host integration, chronic inflammation, and calcific degeneration [33,34,35,36,37]. While minimally invasive and transcatheter approaches have reduced operative trauma and recovery times [38,39], they simultaneously restrict direct tissue access and graft placement, emphasizing the need for biomaterials that are compatible with catheter-based delivery and the dynamic cardiac environment [40].

To address these challenges, emerging strategies have focused on modulating the wound-healing response and supporting tissue repair [14]. Hydrogels, with their injectability, tunable mechanical and biochemical properties, and capacity to deliver therapeutic agents or encapsulate cells, offer a promising platform for improving surgical outcomes [10,11]. By intervening early in the remodeling cascade, hydrogels may mitigate inflammation, reduce fibrosis, promote angiogenesis, and enhance integration between host tissue and implanted materials [18].

### 2.2. Role of Hydrogels in Cardiac Tissue Support

Biomaterials play an essential role in cardiac surgery by reinforcing weakened tissues, sealing defects, and replacing damaged structures. Conventional materials, such as Dacron, polytetrafluoroethylene, and glutaraldehyde-fixed bovine pericardium, offer mechanical durability and ease of use. However, their biological inertness often provokes foreign body reactions, fibrosis, and calcification, which limit their long-term integration with native cardiac tissue [41]. Recent research has focused on developing biointeractive and adaptive materials, particularly hydrogels, that can dynamically interact with the cardiac microenvironment and support functional recovery [7,42].

Hydrogels are uniquely suited to the mechanical and biological milieu of the heart. Their high water content and viscoelastic properties closely mimic those of the native myocardium, enabling them to provide mechanical support in the setting of myocardial infarction (MI) or surgical trauma. For example, injectable borate-crosslinked viscoelastic gelatin hydrogels can match cardiac mechanical dynamics and attenuate fibrosis [43]. By reducing ventricular wall stress and mitigating adverse remodeling, hydrogels help to stabilize the injured myocardium during the vulnerable post-injury phase [13]. In one preclinical study, alginate-based hydrogels injected into an infarcted myocardium preserved wall thickness and limited chamber dilation over several weeks [44].

Beyond passive support, hydrogels serve as highly versatile platforms for the localized and sustained delivery of therapeutic agents. These include growth factors such as vascular endothelial growth factor (VEGF) and insulin-like growth factor 1 (IGF-1), antifibrotic molecules, and gene-edited cell therapies. Common platforms include thermoresponsive hydrogels based on poly(N-isopropylacrylamide) (PNIPAAm) and chitosan-based systems with pH- or temperature-sensitive release profiles, which have been widely explored for controlled delivery in cardiac repair [10,14]. These bioactive cargoes can be released directly into the infarct zone or the surgical bed, thereby enhancing tissue repair while minimizing systemic exposure.

Encapsulating stem cells, including mesenchymal stem cells (MSCs) and induced pluripotent stem cell-derived cardiomyocytes (iPSC-CMs), within hydrogel matrices has shown promise in improving cell retention, viability, and paracrine signaling. Compared to direct injection, hydrogel-mediated cell delivery more effectively promotes angiogenesis, reduces fibrosis, and preserves cardiac contractility in preclinical models [45,46]. Additionally, hydrogel scaffolds can be functionalized with ECM-mimetic motifs (e.g., RGD peptides) or conductive nanomaterials to facilitate cell adhesion, tissue integration, and synchronized electrical conduction across injured myocardial regions [47,48].

Hydrogels have demonstrated their value as anti-adhesion barriers in surgical settings. When applied to the epicardial surface or within the pericardial cavity, bioresorbable hydrogels, such as catechol-functionalized PEG (Ald-AO-Cat) and fibrin hydrogels with collagen-binding peptides, can prevent fibrous tissue bridging between the heart and surrounding structures. This helps to maintain anatomical planes and reduces the risk of injury during reoperation [49,50]. Commercial formulations, such as CoSeal and Seprafilm, have shown early promise in reducing postoperative adhesion severity and are currently being evaluated in cardiovascular surgical contexts [51].

The development of “smart” hydrogels offers new possibilities for cardiac therapy. These materials, such as PNIPAAm-based thermosensitive hydrogels and β-galactosidase-responsive supramolecular hydrogels, can respond to biomechanical strain, enzymatic activity, or changes in pH, enabling on-demand drug release and adaptive mechanical behavior [52,53]. For instance, matrix metalloproteinase (MMP)-sensitive hydrogels degrade selectively in inflamed myocardial zones, allowing site-specific therapeutic action [54]. The integration of emerging technologies, such as 3D bioprinting using gelatin-alginate hydrogels, wearable sensors incorporating flexible conductive materials, and image-guided delivery systems, may further enhance the precision and personalization of hydrogel-based interventions [55,56,57].

Altogether, these innovations underscore the versatility of hydrogels as multifunctional biomaterials capable of addressing diverse challenges in cardiac surgery, from structural support and biological modulation to the prevention of complications and real-time responsiveness (Table 1).

## 3. Types of Hydrogels Used in Cardiac Applications

A wide variety of hydrogels have been developed for cardiac applications and can be broadly categorized into natural, synthetic, and hybrid types. Each class offers distinct advantages and limitations in terms of biocompatibility, mechanical strength, degradation kinetics, and functional customization of the scaffold. Table 2 summarizes the key characteristics and representative materials of these hydrogel types, which are detailed in the following subsections.

### 3.1. Natural Hydrogels: Collagen, Gelatin, Alginate, Fibrin

Natural hydrogels derived from structural proteins or polysaccharides have been extensively explored for cardiac applications because of their intrinsic biocompatibility, biodegradability, and resemblance to the native ECM [58]. These materials facilitate cell adhesion and matrix remodeling via bioactive motifs that interact with integrins and growth factor receptors. The most widely studied natural hydrogels for cardiac use include collagen, gelatin, alginate, and fibrin, each of which offers distinct physicochemical and biological profiles [10].

Collagen, the most abundant ECM protein in the myocardium, supports robust cell adhesion via integrin-binding domains, particularly type I collagen, which enhances cardiomyocyte and endothelial cell viability [59]. It has been applied as both an injectable gel and a sheet-like scaffold for epicardial coverage, facilitating neovascularization and matrix remodeling [60,61]. However, its rapid enzymatic degradation and limited mechanical strength pose challenges under dynamic cardiac loading, often necessitating chemical crosslinking or blending with synthetic polymers to improve its stability [62,63].

Gelatin, a denatured form of collagen, retains key bioactive sequences, such as RGD motifs, and offers a greater chemical versatility. Its methacrylated derivative, gelatin methacryloyl (GelMA), enables photocrosslinkable 3D matrices with tunable stiffness and spatial control [64]. GelMA-based hydrogels have demonstrated efficacy in supporting cell encapsulation, vascularization, and myocardial tissue regeneration in preclinical studies. Nevertheless, their limited mechanical resilience and relatively fast biodegradation may restrict their use in load-bearing cardiac regions [65].

Alginate, a marine-derived polysaccharide, rapidly forms hydrogels upon ionic crosslinking with divalent cations (e.g., Ca^2+^), making it well suited for catheter-based or minimally invasive delivery [66]. Alginate systems have been evaluated as myocardial bulking agents and delivery matrices for stem cells and growth factors in several progressing clinical studies [67,68]. However, native alginate lacks cell-adhesive sequences and often requires peptide functionalization (e.g., RGD) to enhance cellular interactions. Additionally, its in vivo degradation can be inconsistent, requiring careful tuning to align with therapeutic timelines [69].

Fibrin, which is produced by the polymerization of fibrinogen and thrombin, forms a soft, bioresorbable hydrogel that closely resembles the early wound-healing environment. It supports angiogenesis, immune cell recruitment, and scaffold integration [70]. In cardiac applications, fibrin is employed as a transient matrix to deliver growth factors or stem cells during the early phases of repair. However, their rapid enzymatic degradation and insufficient mechanical integrity limit their utility as long-term structural supports [71,72].

In summary, natural hydrogels offer a strong regenerative potential and excellent biocompatibility, making them attractive candidates for cell therapy and localized drug delivery. However, their susceptibility to rapid degradation, mechanical fragility, and batch-to-batch variability poses challenges for applications that require sustained mechanical support and durability. Overcoming these limitations, either through chemical modification or hybridization with synthetic polymers, remains a key focus in adapting natural hydrogels to the dynamic cardiac environment.

### 3.2. Synthetic Hydrogels: PEG, PVA, Polyacrylamide

Synthetic hydrogels have been developed to address the key limitations of natural biomaterials, including variability, insufficient mechanical strength, and rapid biodegradation. Constructed from engineered polymers such as PEG, PVA, and polyacrylamide (PAAm), these systems offer precise control over properties such as stiffness, crosslinking density, degradation kinetics, and chemical functionality [73]. Although inherently bioinert, synthetic hydrogels can be chemically modified to present bioactive ligands or responsive elements, enabling tailored interactions with the cellular microenvironment [74].

PEG-based hydrogels are among the most widely explored synthetic platforms for cardiac applications owing to their hydrophilicity, low immunogenicity, and high tunability [75]. Functional groups, such as acrylate, thiol, and maleimide, can be incorporated to enable crosslinking via photopolymerization or Michael addition. PEG networks are often modified with RGD motifs to promote cell adhesion and with heparin to stabilize growth factors, thereby improving their overall biological functionality [76]. In the cardiac context, PEG hydrogels have been used as anti-adhesion barriers, injectable scaffolds for myocardial support, and carriers for the localized delivery of cells or proteins. However, their lack of intrinsic bioactivity necessitates deliberate modifications to promote cell adhesion, proliferation, and matrix remodeling [50,77].

PVA hydrogels are known to have an excellent mechanical resilience, elasticity, and chemical stability. These features make them attractive for structurally demanding applications, such as heart valve scaffolds and cardiac patches [78,79]. PVA is typically crosslinked through repeated freeze–thaw cycles, forming physically stable networks with low protein adsorption and favorable hemocompatibility. However, the absence of native cell-binding motifs and enzymatic degradability limits their integration with the host tissue, often necessitating blending with natural polymers (e.g., gelatin and chitosan) or the addition of functional groups to improve their biocompatibility [80,81].

PAAm hydrogels offer an exceptional tunability of mechanical properties, and have been widely used to study cardiomyocyte mechanotransduction in vitro. Although concerns regarding monomer toxicity have historically limited their in vivo use, recent advances in copolymer design and purification have improved their safety profile [82]. Notably, nanocomposite PAAm hydrogels incorporating conductive elements such as carbon nanotubes and graphene have shown potential for restoring electrical conductivity and supporting electromechanical coupling across the infarcted myocardium in experimental models [83].

In summary, synthetic hydrogels offer unmatched reproducibility, scalability, and structural control, making them valuable tools for cardiac repair. However, their intrinsic bioinertness remains a significant limitation, often requiring complex modifications to elicit the desired biological responses. This challenge has spurred the development of hybrid hydrogels that aim to integrate the bioactivity of natural polymers with the mechanical precision of synthetic networks, as discussed in the following section.

### 3.3. Hybrid Hydrogels and Smart/Bioresponsive Materials

Hybrid hydrogels have emerged as multifunctional platforms for cardiac applications to bridge the gap between the bioactivity of natural polymers and the mechanical robustness of synthetic matrices. These materials combine biologically derived polymers, such as collagen, gelatin, and hyaluronic acid, with synthetic components, such as PEG and PVA, to create composite systems that balance cellular bioactivity and structural integrity [84,85]. These designs allow for improved control over degradation rates, elasticity, and cell–matrix interactions.

Hybrid hydrogels have demonstrated notable utility in cardiac settings. For example, gelatin–PEG composites form photocrosslinkable networks suitable for in situ gelation, enhancing cell encapsulation, angiogenesis, and mechanical support after myocardial injections [86]. Similarly, alginate–PEG matrices improve cell retention and mitigate inflammatory responses in the infarcted myocardium by establishing a biointeractive and stable environment [87]. These hybrid formulations are also compatible with 3D bioprinting and catheter-based delivery, aligning well with evolving surgical techniques [88].

Building on these foundations, bioresponsive or “smart” hydrogels represent the next generation of adaptive biomaterials. These systems are designed to respond to environmental stimuli, such as pH, temperature, enzymatic activity, and mechanical stress, enabling spatially and temporally controlled drug release or material transformation [89]. Some examples are given as follows:

- pH-sensitive hydrogels release anti-inflammatory agents in acidic ischemic environments, thereby minimizing systemic effects [90].
- MMP-degradable matrices allow for localized degradation and targeted therapeutic delivery in remodeled tissues [54].
- Thermoresponsive hydrogels based on PNIPAAm remain injectable at room temperature and solidify at body temperature, facilitating conformal gelation during minimally invasive procedures [91,92].

A particularly promising innovation involves conductive hydrogels that incorporate nanomaterials such as graphene oxide, polypyrrole, or gold nanoparticles to emulate the electrical properties of the native myocardium. These conductive systems facilitate synchronized electrical signal propagation and electromechanical coupling, which are critical for restoring coordinated cardiac contractions [93]. Preclinical studies have shown improved action potential transmission and contractile synchrony in infarcted heart models using conductive matrices [94].

Finally, the integration of hydrogels into cardiac bioprinting platforms represents a major step toward personalized regenerative therapy. Hydrogels must support not only mechanical stability, but also vascularization and functional tissue maturation. Advanced strategies now include multilayered constructs that combine decellularized cardiac ECM with synthetic polymers to replicate the anisotropic structure and electromechanical properties of the native heart [95]. As hydrogel technologies converge with 3D bioprinting and personalized medicine, the field is moving toward the realization of patient-specific bioelectrically active cardiac grafts that are capable of both structural support and functional restoration.

### 3.4. Crosslinking Mechanisms and Delivery Formats

**Figure 1:**
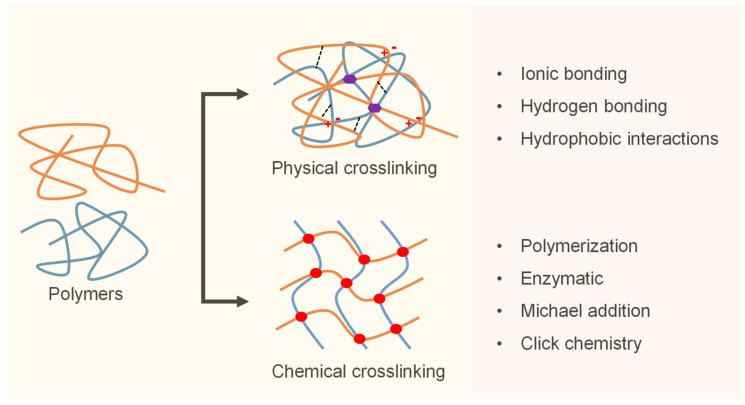
Schematic representation of hydrogel crosslinking mechanisms.

The clinical performance of hydrogels in cardiac surgery is strongly influenced by their crosslinking mechanisms, which govern key parameters such as gelation speed, mechanical integrity, degradation rate, and in vivo retention [18]. Crosslinking strategies are broadly classified into physical and chemical mechanisms, each offering distinct advantages depending on the procedural requirements and biological context (Figure 1) [96].

Physically crosslinked hydrogels rely on non-covalent interactions, such as ionic bonding, hydrogen bonding, and hydrophobic associations. These systems are typically shear-thinning, reversible, and capable of rapid gelation, making them ideal for catheter-based or injectable delivery [97]. A well-known example is calcium-crosslinked alginate, which gels upon exposure to tissue-resident divalent cations, allowing for minimally invasive administration without external triggers [98]. However, its relatively low mechanical strength can limit durability under repetitive loading of the beating heart unless supplemented with secondary stabilization strategies [99].

In contrast, chemically crosslinked hydrogels form covalent bonds that offer a superior mechanical stability and controlled degradation. Common crosslinking chemistries include photopolymerization, Michael addition, enzymatic catalysis (e.g., horseradish peroxidase and transglutaminase), and click reactions such as thiol–ene and azide–alkyne cycloadditions [100]. These systems enable fine-tuned control over gelation kinetics and structural properties, facilitating precise deployment in dynamic cardiac environments. Recent advances in light-activated systems using visible or near-infrared wavelengths have improved intraoperative usability and cytocompatibility [101].

To integrate the strengths of both approaches, dual-crosslinking systems have been developed. These hydrogels undergo initial physical gelation to allow for easy injection, followed by secondary chemical crosslinking in situ to ensure long-term retention and biomechanical compliance [102]. Such modular systems are particularly valuable in cardiac surgery, where timing, tissue adhesion, and mechanical adaptation must closely align with procedural constraints [103].

The hydrogel delivery format is another critical determinant of clinical success. Depending on the surgical context, hydrogels may be applied as preformed patches during open procedures, sprayed onto exposed cardiac surfaces, or injected via a needle or catheter in minimally invasive or robotic-assisted settings [18]. In situ-forming hydrogels that polymerize in response to physiological stimuli, such as pH, enzymatic activity, or temperature, offer enhanced adaptability to irregular cardiac anatomy and facilitate site-specific therapy [104]. Important design considerations include gelation time, optical visibility during surgery, and compatibility with co-delivered agents, such as cells, growth factors, and nanoparticles.

In summary, the clinical translation of hydrogel systems to cardiac applications depends on the seamless integration of crosslinking chemistry, material architecture, and delivery strategy. Achieving clinical success will depend on co-optimizing these design variables, not only to meet biological performance metrics, but also to ensure seamless integration into evolving surgical workflows.

### 3.5. Design Criteria for Hydrogels in Cardiac Surgery

To function effectively in the cardiac environment, hydrogels must meet several key physicochemical and mechanical criteria. These include the following:

- Biomechanical compatibility, such as elastic modulus and viscoelastic behavior, which approximates native myocardial tissue to avoid mechanical mismatch and support functional integration [59,86].
- Controlled degradation, allowing the material to persist throughout the therapeutic window (typically 2–4 weeks) before resorption, synchronized with tissue healing dynamics [69,72,92].
- Tissue adhesion and retention, particularly under dynamic epicardial conditions, necessitating chemical or physical strategies to enhance hydrogel anchoring without inducing inflammation [76,93].
- Injectability and in situ gelation, enabling minimally invasive delivery via catheter or needle followed by solidification upon exposure to physiological conditions such as temperature, ions, or enzymes [66,98].
- Biocompatibility and minimal immunogenicity, often achieved through the use of ECM-mimetic materials or functionalized synthetic scaffolds [59,74].
- Functionalization capacity, such as incorporating growth factors, cells, or responsive moieties for bioactivity and adaptive behavior [87,89].

Together, these properties form the foundation for the rational design of cardiac hydrogels. Optimizing these interrelated variables is essential to ensure not only biological efficacy, but also clinical usability across diverse surgical workflows.

## 4. Myocardial Repair and Regeneration

MI causes irreversible cardiomyocyte loss and fibrotic scarring, leading to progressive ventricular remodeling, contractile dysfunction, and heart failure [105]. Owing to the limited regenerative capacity of the heart, effective myocardial repair remains an unmet clinical challenge.

Hydrogels have emerged as promising platforms for myocardial regeneration owing to their biocompatibility, mechanical tunability, and ability to localize therapeutic interventions at the site of injury [13]. These materials provide biomechanical support while delivering stem cells, growth factors, immunomodulators, and gene therapies to modulate the post-infarction microenvironment.

### 4.1. Hydrogel-Based Cardiac Patches

Epicardial hydrogel patches have gained traction as dual-function systems for cardiac repair, offering both mechanical reinforcement and localized therapeutic delivery [13]. In a preclinical study using a rat MI model, a PEG–fibrinogen composite patch loaded with VEGF was applied to the epicardium. Four weeks post-implantation, the treated animals demonstrated improved ejection fraction (EF), enhanced neovascularization, and reduced infarct expansion. These effects were attributed to the synergistic impact of biomechanical stabilization and sustained VEGF release [106].

To enhance electrophysiological integration, conductive nanomaterials such as carbon nanotubes and polypyrrole have been incorporated into hydrogel matrices [107]. In one study, a gelatin-based patch embedded with single-walled carbon nanotubes and seeded with cardiomyocytes promoted connexin-43 expression and improved action potential propagation in the infarcted myocardium. Treated hearts showed an enhanced contractile performance and reduced ventricular dilation, suggesting functional restoration via bioelectrical coupling [108].

Beyond their biological functions, hydrogel patches provide mechanical stabilization by thickening the infarct region, lowering wall stress, and preventing pathological remodeling. This helps to preserve left ventricular geometry and mitigates the progression of heart failure [109].

In summary, epicardial hydrogel patches act as multifunctional constructs that reinforce the damaged myocardium, deliver regenerative cues, and facilitate electromechanical recovery when integrated with conductive materials. Their versatility makes them a compelling platform for structural and functional restoration after MI.

### 4.2. Stem Cell Encapsulation and Retention

The poor engraftment and survival of transplanted cells remain major barriers to the success of cell-based cardiac therapy. Transplanted cells are frequently lost because of mechanical washout, oxidative stress, and an inflammatory post-infarction environment [110]. Injectable hydrogels offer a promising solution by providing a supportive 3D matrix that enhances cell retention, reduces apoptosis, and sustains the release of paracrine factors. Their viscoelasticity allows for minimally invasive delivery while preserving spatial localization [111].

In a rat MI model, IL-10-overexpressing MSCs encapsulated in a fibrin hydrogel significantly reduced fibrosis and enhanced vascular density compared to MSCs alone, highlighting the synergistic effects of hydrogel-mediated retention and immunomodulation [112]. Similarly, a thermoresponsive gelatin hydrogel (Col-Tgel) improved MSC viability and engraftment, resulting in superior preservation of ventricular function in a murine model of infarction [113].

Hydrogels also support the delivery of iPSC-CMs. A 3D bioprinted scaffold composed of GelMA and type I collagen maintained iPSC-CM viability, preserved the sarcomere structure, and supported calcium handling. Notably, when disease-specific iPSC-CMs from patients with catecholaminergic polymorphic ventricular tachycardia were embedded in this hydrogel, they responded appropriately to adrenergic stimulation, demonstrating dual functionality as both a therapeutic platform and a disease model [114]. This was further validated in a porcine model, where the injection of a hydrolyzed gelatin hydrogel with iPSC-CMs increased myocardial wall thickness and EF after four weeks [45].

Collectively, these findings highlight the potential of hydrogels to overcome the key limitations of stem cell therapy by enhancing cellular retention, prolonging paracrine signaling, and promoting integration with the host myocardium. As hydrogel formulations become increasingly biomimetic and tunable, their role in durable myocardial regeneration is likely to expand.

### 4.3. Angiogenic and Immunomodulatory Hydrogels

Adequate vascularization and regulated immune responses are essential for effective myocardial regeneration, as they ensure sustained oxygen and nutrient delivery while preventing maladaptive fibrosis after infarction [115]. Hydrogels can be engineered to deliver pro-angiogenic factors, such as VEGF, basic fibroblast growth factor (bFGF), and stromal cell-derived factor-1α, which stimulate endothelial proliferation and capillary network formation [116]. In MI models, gelatin- and alginate-based hydrogels loaded with these cytokines consistently promoted neovascularization and preserved systolic function. For instance, the application of a bFGF-loaded gelatin hydrogel in a chronic rat MI model significantly increased capillary density, improved left ventricular contractility, and reduced the expression of fibrosis-related genes [117]. Similarly, an alginate hydrogel co-delivering VEGF-A_165_ and platelet-derived growth factor-BB induced the robust formation of α-smooth muscle actin (SMA)-positive vasculature and sustained ventricular output, as evidenced by elevated systolic velocity time integrals [118].

In addition to promoting angiogenesis, modulating the post-infarction inflammatory response is crucial for guiding myocardial healing toward regeneration rather than fibrosis. Prolonged or excessive inflammation after ischemic injury can impair matrix remodeling and functional recovery [119]. To address this, an alginate hydrogel delivering Annexin A1, a pro-resolving mediator, significantly reduced macrophage infiltration and collagen deposition while promoting neovascularization in a murine MI model, shifting the repair trajectory toward regeneration [120]. An alternative strategy involved a supramolecular NapFFY hydrogel co-delivering the NF-κB inhibitor SN50 and IL-10, which was tested in a rat MI model. This dual-release system not only polarized macrophages toward an anti-inflammatory M2 phenotype, but also suppressed pro-inflammatory cytokines, enhanced angiogenesis, and markedly improved cardiac remodeling and function by day 28 post-infarction [121].

To further enhance therapeutic efficacy, recent studies have focused on multifunctional hydrogels that integrate angiogenic, immunomodulatory, and conductive properties. For example, MXene–gelatin composite patches restored impaired electrical conduction in the infarcted myocardium by upregulating connexin-43 and improving contractile performance [122]. Another sophisticated system combined miR-21-5p-loaded mesoporous silica nanoparticles within a pH-responsive GelMA-based hydrogel (Gel@MSNs/miR-21-5p). When injected into a porcine MI model, this formulation promoted angiogenesis via VEGF/ERK pathway activation while concurrently dampening inflammation through toll-like receptor (TLR) 2/NF-κB inhibition. Treated hearts exhibited a reduced infarct size, increased capillary density, and significantly improved global cardiac function [123].

These studies highlight the capacity of hydrogels to simultaneously promote vascular growth, modulate immune responses, and restore electromechanical function. With the continued evolution of hydrogel technologies, multifunctional systems hold significant promise for comprehensive myocardial repair.

### 4.4. Preclinical Models and Efficacy Outcomes

The clinical translation of hydrogel-based therapies to myocardial repair relies on a consistent efficacy across preclinical models and early-phase trials. Rodent studies have demonstrated that hydrogels that deliver stem cells or bioactive factors improve infarct wall thickness, enhance EF, and promote neovascularization. A meta-analysis reported mean EF increases of approximately 9% in rats and 16% in mice, along with improved fractional shortening [124]. In one representative study, a collagen–PEG hydrogel encapsulating bone-marrow-derived MSCs enhanced EF, increased wall thickness, and reduced infarct size in rats compared to PBS controls, findings attributed to improved MSC retention and paracrine activity [125].

To support translational relevance, large animal models, such as swine models, have been used to evaluate delivery feasibility and therapeutic durability. In a porcine model, the intracoronary injection of calcium-crosslinked alginate four days post-MI significantly increased scar wall thickness by 53% and reduced ventricular dilation relative to saline-treated controls using catheter-based techniques similar to those used in human interventions [44].

Building on these findings, early human trials have confirmed the feasibility and safety of hydrogel therapy for ischemic heart disease. In a first-in-human randomized trial, 27 patients with ST-elevation MI received intracoronary IK-5001 (calcium-crosslinked alginate hydrogel) within seven days of infarction. The procedure was well tolerated, with no serious device-related events or arrhythmias reported over 6 months. Compared to placebo, the treated group showed attenuated ventricular dilation and preserved EF on echocardiography, supporting the structural benefits [126].

In the chronic MI setting, a phase I trial tested the transendocardial injection of VentriGel, a decellularized myocardial ECM hydrogel, in patients from 2 months to 3 years post-infarction. The treatment was safe, and exploratory endpoints showed an improved 6-min walk distance, New York Heart Association (NYHA) class, and trends toward favorable ventricular remodeling [127].

Among the most clinically advanced systems, Algisyl-LVR is an injectable alginate hydrogel administered via intramyocardial injection through limited thoracotomy. It functions as a mechanical bulking agent, increasing wall thickness and reducing myofiber stress to counteract adverse remodeling effects. Patient-specific finite element modeling has demonstrated reduced end-systolic and end-diastolic stress and improved ventricular geometry and function [128].

These mechanisms translated into clinical benefits in the AUGMENT-HF trial, a multicenter randomized study of 78 patients with advanced heart failure. Compared to medical therapy alone, Algisyl-LVR recipients experienced significant improvements in peak VO_2_, 6-min walk distance, and NYHA class, all of which were sustained at 12 months. No increase in serious adverse events was observed, and echocardiography confirmed reverse remodeling [129,130].

The results of AUGMENT-HF suggest that hydrogel-mediated structural augmentation represents a novel therapeutic paradigm distinct from pharmacological or cell-based approaches by directly modulating biomechanical stress. A follow-up trial (AUGMENT-HF II) is ongoing to evaluate long-term outcomes, including mortality and rehospitalization rates [131].

Collectively, preclinical and clinical studies have underscored the multifaceted therapeutic potential of hydrogel systems for myocardial repair. By providing mechanical support, modulating fibrosis, and enabling the targeted delivery of regenerative cues, these materials address key pathophysiological mechanisms in both ischemic and non-ischemic heart failure. Injectable scaffolds and epicardial constructs, such as Algisyl-LVR, exemplify the feasibility of biomaterial-based structural cardiac therapy. Future directions should emphasize material optimization, tunable degradation, and minimally invasive delivery, along with large-scale clinical trials to validate long-term safety and efficacy. These strategies are summarized in Table 3, which highlights the diverse hydrogel-based approaches for myocardial repair and their therapeutic outcomes.

## 5. Pericardial and Adhesion Barriers

Postoperative pericardial adhesions are common and clinically significant complications of cardiac surgery. These fibrotic bands form between the epicardium and surrounding structures, such as the sternum, pleura, or pericardial remnant, particularly after pericardiotomy, trauma, or exposure to foreign materials. In reoperative settings, dense adhesions significantly increase the risk of intraoperative bleeding, myocardial laceration, or injury to bypass grafts and great vessels, often resulting in prolonged operative times and worsened surgical outcomes [32].

As staged and repeat cardiac surgeries become increasingly common, especially in pediatric congenital heart disease and adult reoperative coronary or valvular procedures, adhesion prevention has become a critical surgical priority [51].

Hydrogel-based barriers have gained increasing attention because of their ability to conform to irregular cardiac surfaces, predictably degrade, and elicit minimal inflammatory responses. These features make them well-suited as temporary physical barriers that prevent tissue contact during the early postoperative period, when adhesions are most likely to develop [132]. Consequently, hydrogels are being actively investigated as next-generation anti-adhesion agents for both open and minimally invasive cardiac procedures.

### 5.1. Pathophysiology of Post-Surgical Adhesions

Pericardial adhesion formation is a multifactorial process triggered by surgical trauma to the pericardium. Following pericardiotomy, the disruption of mesothelial integrity initiates an acute inflammatory cascade involving increased vascular permeability, neutrophil and macrophage infiltration, and the release of pro-inflammatory cytokines. This inflammatory response leads to the accumulation of fibrin-rich exudates within the pericardial cavity, forming a provisional matrix for adhesion development. In the absence of effective fibrinolysis, the fibrin scaffold is colonized by fibroblasts and myofibroblasts, resulting in excessive collagen deposition and the formation of vascularized fibrotic adhesions [32].

Multiple perioperative factors can exacerbate this pathological cascade, including mechanical manipulation, hemorrhage, ischemia–reperfusion injury, and exposure to foreign materials such as synthetic patches, prosthetic valves, and suture materials. These stimuli amplify local inflammation and promote aberrant tissue remodeling. Over time, adhesions mature into dense contractile bands that tether the heart to the surrounding structures, restrict pericardial mobility, and complicate surgical re-entry [133].

Reoperative cardiac surgery in the presence of established adhesions is associated with a significantly increased intraoperative risk of myocardial laceration, bypass graft injury, and vascular damage. These risks are particularly pronounced in pediatric congenital heart disease and in adult patients undergoing repeat coronary or valvular procedures [134]. This clinical challenge underscores the need for bioresorbable barrier materials that can be applied intraoperatively to temporarily separate tissue surfaces. Ideally, these materials should prevent early adhesion without impairing normal healing, allow complete resorption, and preserve cardiac function.

### 5.2. Hydrogel-Based Anti-Adhesion Barriers

**Figure 2:**
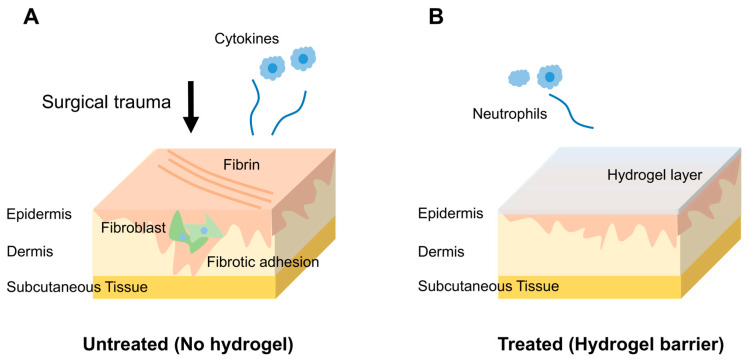
Schematic representation of hydrogel-based anti-adhesion strategies. (**A**) Untreated (No hydrogel); (**B**) Treated (Hydrogel barrier).

Hydrogels have emerged as promising candidates for preventing pericardial adhesion because of their unique combination of biocompatibility, controlled degradation kinetics, and capacity to conform to the dynamic and irregular surface of the beating heart [135]. When applied intraoperatively, these materials function as temporary physical barriers that prevent tissue apposition during the critical early postoperative window, typically within 2–3 weeks, when adhesions most commonly develop. Unlike conventional anti-adhesion agents, hydrogels accomplish this without provoking foreign body reactions or impairing myocardial function (Figure 2) [132].

Various hydrogel formulations have demonstrated efficacy in preclinical models. One example is the thermosensitive poloxamer–alginate–calcium chloride hydrogel (PACM), which undergoes a sol–gel transition at body temperature, enabling facile application and rapid in situ gelation. In a rabbit pericardiotomy model, PACM significantly reduced the gross adhesion area and fibrosis scores compared to saline controls. Although the levels of inflammatory markers tended to be lower, statistical significance was not achieved, indicating only modest anti-inflammatory effects [136].

Another system, AdSpray, a dextrin-based hydrogel, was evaluated in a rabbit model of intrapericardial adhesion. Treatment significantly reduced both the extent and severity of adhesions. Histological analysis revealed fewer BrdU-positive proliferating cells in the epicardium, suggesting attenuation of the injury-induced proliferative response. The material was fully resorbed by day 7, confirming its short-term bioresorbability; however, further studies are required to clarify its degradation kinetics and long-term cardiac safety [137].

PEG-based hydrogels functionalized with catechol and aminooxy groups, which mimic mussel adhesive proteins, have been developed to enhance the mechanical stability of contracting epicardial surfaces. These catechol–oxime hydrogels demonstrated strong wet-tissue adhesion and outperformed commercial agents in porcine models, significantly reducing adhesion area and severity. Mechanistic studies have attributed their efficacy to both physical separation and reduced fibrosis and inflammation, underscoring the importance of surface chemistry in modulating local biological responses [50].

Beyond passive separation, next-generation hydrogels are engineered with intrinsic bioactivity to actively influence the pericardial wound-healing response. For example, a photocrosslinkable GelMA–dopamine/silk fibroin hydrogel exhibited stable adhesion to the epicardium under dynamic conditions. In a rat pericardial adhesion model, this system completely prevented adhesion formation by postoperative day 14. Molecular analyses revealed reduced collagen deposition, α-SMA expression, and levels of pro-inflammatory cytokines, such as IL-6 and TGF-β1, indicating the robust suppression of both fibrotic and inflammatory pathways [138].

A key determinant of anti-adhesion efficacy is the degradation profile of the hydrogel. The material must persist throughout the acute inflammatory phase, but degrade in a timely manner to avoid hindering tissue integration or inducing chronic inflammation [132]. This degradation window can be precisely tuned by modifying the polymer crosslinking density, hydrophilicity, and chemical structure. For instance, increasing the ester content or incorporating hydrolytically labile linkers accelerates breakdown under physiological conditions [50].

The delivery format also plays a crucial role in intraoperative feasibility. Injectable and sprayable hydrogels are particularly advantageous in minimally invasive and robotic procedures, where access to the surgical field is restricted. These formats enable rapid and uniform application to the beating heart and ensure consistent barrier application with minimal operator variability [139].

Together, these design and delivery considerations will guide the development of clinically viable hydrogel barriers. By integrating tunable degradation, robust epicardial adhesion, and biological functionality, these systems offer a multifaceted solution to one of the most persistent challenges in cardiac surgery.

### 5.3. Clinical Translation of Hydrogel Barriers

Although numerous hydrogel formulations have demonstrated efficacy in animal models, few have advanced to human studies in cardiac surgery, and none have received regulatory approval for this indication [51]. Representative hydrogel-based anti-adhesion systems differ in their material composition, crosslinking strategy, delivery format, and degradation kinetics. Table 4 summarizes selected commercial and investigational products relevant to cardiac applications.

Some anti-adhesion products currently used in clinical practice, such as Seprafilm, a hyaluronic acid–carboxymethylcellulose film, were originally designed for abdominal or pelvic procedures and are occasionally used off-label in cardiac operations. Retrospective analyses of pediatric patients undergoing re-sternotomy for congenital heart disease reported that Seprafilm application during the initial surgery was associated with shorter dissection times and reduced adhesion tenacity without increasing the risk of infection or bleeding [140]. Similarly, prospective studies have shown less tenacious adhesions with its use [141]. However, Seprafilm’s poor adherence to moist, beating cardiac tissue and its rapid degradation within 7 days limit its effectiveness during the 2–3-week postoperative adhesion window.

Among cardiac-adapted materials, CoSeal, a sprayable PEG-based hydrogel, has shown encouraging results. In a multicenter observational study conducted across seven European centers, CoSeal was applied to the epicardium and great vessels of 76 pediatric patients undergoing staged congenital heart surgeries. Among the 36 patients who underwent reoperation ≥3 months post-application, 85% exhibited filmy and avascular adhesions and 57% demonstrated a mild profile across all anatomical regions. Surgeons reported improved re-entry conditions and satisfactory handling. Although early use was associated with six adverse events (e.g., tamponade and arrhythmia), subsequent weight-based dosing mitigated these issues, and no further hydrogel-related complications were observed [142].

Together, these early clinical findings suggest that hydrogel-based adhesion barriers may enhance surgical outcomes during cardiac reoperation. However, current formulations remain suboptimal, particularly in terms of mechanical persistence and adherence to dynamic epicardial surfaces. Advancing hydrogel barriers for routine cardiac use will require not only improved material design, ensuring mechanical durability and epicardial adherence, but also robust validation through well-powered prospective clinical trials.

### 5.4. Regulatory and Technological Outlook

To date, no hydrogel-based anti-adhesion barrier has received regulatory approval for use in cardiac surgery. These products are typically classified as Class III medical devices, requiring comprehensive evidence of their safety and clinical efficacy [143]. In the cardiac setting, additional scrutiny is applied because of the proximity of critical structures and the potential for adverse events, such as pericardial effusion, arrhythmogenesis, and interference with pacemaker leads and conduction pathways. Consequently, parameters such as long-term biocompatibility, degradation kinetics, local immune modulation, and electrical inertness have been prioritized in regulatory reviews [51,144].

To address these demands, next-generation hydrogel systems are being designed to function not only as physical barriers, but also as active modulators of the wound-healing microenvironment. Recent advances include the development of thermoresponsive polymers that undergo rapid in situ gelation, antifouling surfaces that minimize protein adsorption and cellular attachment, and bioadhesive chemistry engineered to accommodate dynamic epicardial motion. For instance, sprayable thermoresponsive hydrogels with fouling-resistant characteristics have demonstrated stable adherence to the porcine epicardium, maintaining structural integrity throughout the critical 2–3-week adhesion-forming period and degrading without eliciting inflammatory responses [139]. Likewise, catechol-functionalized PEG–oxime hydrogels have achieved ultrafast gelation (<3 s), low swelling ratios, and prolonged epicardial retention. In preclinical models, these systems significantly reduced adhesion severity compared to conventional barriers [50].

As minimally invasive and robot-assisted cardiac procedures become increasingly common, the demand for hydrogel systems that are compatible with catheter-based or endoscopic delivery is growing. In response, hybrid constructs that integrate fibrin-based hydrogels with decellularized pericardial matrices have been developed to facilitate spray application and improve mechanical anchoring. In porcine reoperative models, these formulations demonstrated strong epicardial adherence and a marked reduction in the severity of adhesions at surgical re-entry [49].

Ultimately, the clinical viability of hydrogel-based barriers depends on their ability to meet procedural demands, actively modulate pericardial healing, and yield consistent outcomes in diverse patient populations. Achieving regulatory approval will require rigorous randomized clinical trials that demonstrate not only safety, but also durable efficacy in preventing adhesions. As the frequency of staged and reoperative cardiac interventions continues to increase, the development and adoption of these materials are essential to mitigate surgical risks and improve the safety and efficacy of reoperative cardiac care.

To expedite the transition of hydrogel systems into advanced clinical trials and regulatory approval, early collaboration with regulatory agencies is essential [143]. This includes aligning preclinical endpoints with clinically meaningful outcomes, incorporating risk-based classifications, and leveraging accelerated programs such as Breakthrough Device or PRIority MEdicines (PRIME) designations [144]. Adaptive trial designs, particularly those integrating imaging or biomarker-based endpoints, may help to capture the multifaceted benefits of hydrogel therapies while minimizing sample size and cost [145].

However, even with these strategies in place, several practical and systemic challenges remain. These include variability in hydrogel synthesis and application protocols across clinical sites, the limited standardization of delivery methods (e.g., open surgery vs. catheter-based approaches), and the lack of validated surrogate markers for efficacy, particularly in adhesion prevention or myocardial support. Additionally, successful integration into surgical workflows requires seamless coordination across multidisciplinary teams, including surgeons, anesthesiologists, and biomaterials specialists [51]. Overcoming these translational barriers, including variability in protocols, limited standardization, and lack of interdisciplinary integration, is critical to fully realize the clinical potential of hydrogel-based interventions in cardiac surgery.

## 6. Valve and Patch Engineering

Hydrogels are increasingly recognized as versatile and biologically responsive materials for cardiac tissue engineering, particularly for developing tissue-engineered heart valves (TEHVs) and myocardial repair patches. While conventional prosthetic valves have markedly improved survival in patients with valvular heart disease, they are associated with several limitations, including thrombogenicity, structural degeneration over time, and a lack of growth potential. These shortcomings are especially problematic in pediatric populations, where ongoing somatic growth necessitates long-term adaptability and device scalability [146].

Similarly, patches used to repair congenital or acquired cardiac defects, whether synthetic (e.g., expanded polytetrafluoroethylene) or biologic (e.g., decellularized pericardium), often result in complications such as calcification, immune rejection, or insufficient integration with the highly dynamic myocardial tissue [147].

Hydrogels offer compelling advantages in these contexts owing to their excellent biocompatibility, tunable mechanical properties, and high water content that mimics the ECM of native cardiac tissue. These properties support cellular viability, infiltration, and tissue remodeling, which are critical elements for achieving functional integration. Accordingly, hydrogels are emerging as next-generation scaffolds capable of overcoming both the biological and biomechanical limitations of traditional implant materials [10]. Representative examples of hydrogel-based strategies for valve and patch engineering are summarized in Table 5.

### 6.1. Hydrogel Scaffolds for Valve Leaflets

TEHVs aim to replicate the biomechanical functionality and biological adaptability of native valve leaflets. Achieving this goal requires scaffolds that facilitate host cell infiltration, ECM deposition, and synchronized degradation during tissue regeneration. Hydrogels have emerged as attractive scaffolding materials owing to their high water content, tunable stiffness, and viscoelastic behavior, which closely mimic the mechanical milieu of native cardiac ECM. These properties enable dynamic cell–matrix interactions and help to preserve the quiescent phenotype of valvular interstitial cells (VICs), a key factor in preventing fibrosis and maintaining leaflet compliance [148].

One notable strategy involves a photocrosslinkable hydrogel composed of methacrylated hyaluronic acid and GelMA. This dual-polymer system leverages the mechanical strength of hyaluronic acid and bioactivity of gelatin to create a permissive microenvironment for VIC viability and function. In vitro experiments have shown that encapsulated VICs remain highly viable and retain a quiescent fibroblast-like phenotype characterized by low α-SMA expression. This phenotype is critical for preserving valve elasticity and minimizing maladaptive fibrotic remodeling. Additionally, hydrogels support the spatially organized deposition of key ECM proteins, including type I collagen and elastin. By modulating scaffold stiffness via photopolymerization, researchers have influenced cell morphology and matrix alignment, highlighting the potential of scaffolds to direct tissue architecture during regeneration [149].

To enhance biointegration, some hydrogel formulations have incorporated native ECM components. For example, PEG-diacrylate hydrogels embedded with microparticles derived from decellularized porcine valve tissue have demonstrated improved VIC adhesion, infiltration, and ECM synthesis. These composite scaffolds also supported leaflet-like motion under pulsatile flow in a bioreactor setting. When endothelial cells were seeded onto the luminal surface, they formed a confluent CD31-positive monolayer, indicating the re-establishment of a non-thrombogenic endothelium, which is an essential criterion for long-term valve durability [150].

Additional innovations have focused on engineering hydrogel scaffolds that are responsive to mechanical stimuli, such as shear stress, to better replicate the dynamic hemodynamic environment of native valves. For instance, collagen–hyaluronic acid hydrogels have been shown to promote endothelial cell elongation and alignment in response to physiological shear, mediated by cytoskeletal rearrangements and microtubule acetylation [151]. Although not yet tested in valve-specific contexts, RGD-functionalized alginate hydrogels have demonstrated a similar endothelial responsiveness under laminar flow, supporting their potential for future applications in mechanically adaptive valve scaffolds [152].

Together, these studies underscore the potential of hydrogel-based scaffolds in TEHV development. By offering mechanical support, bioinstructive cues, and responsiveness to mechanical forces, these materials are poised to enable next-generation valve replacements that integrate seamlessly with the host tissue and adapt to physiological demands over time.

### 6.2. Mechanical and Hemodynamic Requirements

A major challenge in hydrogel-based heart valve design is the inherently low mechanical strength and viscoelasticity of most formulations. Native aortic valve leaflets exhibit circumferential elastic moduli in the range of 4–10 MPa and ultimate tensile strengths of approximately 1.5–2.5 MPa, depending on the layer and loading direction [153]. These mechanical demands are further compounded by the fact that valve leaflets undergo over 100,000 cycles of high-amplitude mechanical stress per day, necessitating scaffolds that are not only biocompatible, but also capable of withstanding cyclic strain without structural fatigue or failure [148]. To meet these demands, hydrogels must be reinforced using composite strategies or hybridized with mechanically robust components [154].

A widely adopted reinforcement approach involves the incorporation of nanofibers or particulate fillers into the hydrogel matrix to enhance its mechanical properties while maintaining cytocompatibility. For instance, interpenetrating networks composed of GelMA and pectin-grafted polycaprolactone (PCL) have demonstrated significant improvements in compressive moduli, increasing from <0.1 MPa (GelMA alone) to approximately 5 MPa without adversely affecting cell viability [155]. Similarly, coaxial nanofiber composites combining PCL with GelMA embedded within bulk hydrogels have shown an enhanced tensile strength (from ~0.2 MPa to ~2.0 MPa) and compressive resilience (from ~0.1 MPa to ~1.5 MPa), sustained shape recovery under cyclic loading, and preserved cell viability [156]. Although these systems have not yet been specifically applied in valve engineering, they represent promising platforms for achieving the mechanical durability required for leaflet scaffolds.

Beyond bulk mechanical reinforcement, functional valve constructs must replicate appropriate hemodynamic behavior. This includes a transvalvular pressure gradient below 10 mmHg, an effective orifice area of ≥1.5–2.0 cm^2^ depending on valve size, a regurgitant fraction of <10%, and smooth flow profiles with low turbulence [157]. Effective leaflet motion requires rapid and complete opening and closure under physiological pressure gradients, with minimal regurgitation and low transvalvular resistance. To address these performance criteria, hydrogel–fiber composites with anisotropic mechanical properties have been developed. For example, PEG-based hydrogels reinforced with electrospun PCL fibers exhibited circumferential elastic moduli of approximately 3.8 MPa, promoted VIC alignment, and preserved leaflet geometry under dynamic flow-loop cycling [158]. In more advanced designs, 3D-bioprinted tri-leaflet valves composed of PEG–PCL–dopamine acrylate hydrogels mounted on stent frames achieved functional opening and closure in pulsatile bioreactor systems. While these constructs maintained low regurgitant volumes, minor malcoaptation at elevated flow rates suggests the need for the further refinement of leaflet geometry and attachment interfaces [159].

An additional biomechanical consideration is the recreation of regional heterogeneity, which is a defining feature of the native aortic valve architecture. Natural valve leaflets exhibit spatial gradients in collagen and elastin fiber alignment, imparting direction-dependent stiffness and complex deformation patterns. To emulate this structural complexity, advanced fabrication techniques, such as photolithographic stiffness patterning and magnetic alignment of anisotropic fillers, have been proposed. These methods enable the spatial tuning of mechanical properties within hydrogel matrices, thereby allowing for more biomimetic structure–function integration [160,161]. In vascular applications, hydrogels may also be engineered to respond to physiological shear stress, typically ranging from ~0.1–0.7 Pa in veins to ~1–2 Pa in arteries [162]. Matching this range can support endothelial alignment, barrier integrity, and native-like flow responsiveness in hydrogel constructs.

In summary, although hydrogels offer a highly favorable environment for cellular viability and biointegration, their intrinsic mechanical limitations necessitate rational composite engineering and precise fabrication techniques. Achieving both mechanical robustness and hemodynamic functionality is essential for the successful clinical translation of hydrogel-based valve scaffolds.

### 6.3. Hybrid Constructs and 3D Printing Approaches

To overcome the mechanical and functional limitations of standalone hydrogels, hybrid constructs that integrate hydrogels with synthetic polymers, ECM components, or supportive scaffolds have been developed. These composite systems aim to synergize the biological advantages of hydrogels, such as their bio-instructive properties, biocompatibility, and high water content, with the mechanical robustness and structural tunability of synthetic materials [148]. Through careful design, hybrid constructs can provide the mechanical integrity required for cardiac loading while maintaining a microenvironment that is conducive to cell infiltration, remodeling, and integration.

A representative example involves the fabrication of electrospun tri-leaflet scaffolds composed of PCL and poly(L-lactic acid) mounted on metallic stent frames and seeded with porcine VICs and cardiac stem cells. These constructs demonstrated effective opening and closure under pulsatile flow, with a hemodynamic performance, including orifice velocity and leaflet excursion, comparable to that of a commercially available Edwards 2800 valve in pulse duplicator testing. However, these evaluations were conducted under right-sided pressure conditions representative of pulmonary circulation (~20 mmHg), and their performance under systemic arterial pressure remains to be validated [163].

Three-dimensional bioprinting has also emerged as a transformative approach for fabricating anatomically accurate and patient-specific cardiac constructs. Multi-nozzle extrusion systems enable the spatial patterning of hydrogel-based bioinks, allowing for the precise engineering of tri-leaflet aortic valve models. One notable study used GelMA–alginate blends to print leaflet constructs that exhibited a high cell viability, structural fidelity, and physiological motion under simulated flow. By tuning the crosslinking density and print orientation, researchers have achieved region-specific stiffness gradients that mimic the mechanical anisotropy of native valve tissue [164].

These bioprinting strategies have been extended to the development of myocardial patches. Using alginate–gelatin bioinks, constructs containing cardiomyocytes, endothelial cells, and fibroblasts were printed into contractile cardiac tissues with an aligned architecture. These engineered strips exhibited synchronized beating, electrical coupling, and early signs of angiogenic sprouting, which are key features for integration with the host myocardium in future clinical applications [165]. The ability to incorporate multiple cell types into spatially defined architectures highlights the potential of 3D printing to generate vascularized and functional cardiac grafts.

Despite these advances, the clinical translation of hydrogel-based valves and myocardial patches remains limited by several factors. These include ensuring long-term durability under physiological hemodynamic loads, achieving immune compatibility, and navigating complex regulatory classifications, particularly for constructs that combine degradable materials with living cells. Moreover, interactions between hydrogel-based constructs and host tissues in high-pressure environments, such as the left ventricle or aortic root, require thorough validation in large animal models before human application [166,167].

Preclinical studies of hydrogel-based heart valves have demonstrated functional performances from over several weeks to months in large animal models [168]. However, achieving a clinically durable lifespan, typically defined as over 15 years for adult patients and at least 10 years for pediatric populations, remains a major challenge and an active focus of biomaterial innovation [169].

Nonetheless, the modularity, tunability, and printability of hydrogels continue to position them at the forefront of cardiac tissue engineering. Advances in materials science, biofabrication, and cell–matrix interaction control have enabled the production of next-generation constructs tailored to patient-specific needs, capable of in situ remodeling, and functional under dynamic mechanical conditions. These technologies offer promising pathways toward scalable and clinically translatable solutions for heart valve replacement and myocardial repair [58].

## 7. Drug and Gene Delivery via Hydrogels

The myocardium presents a uniquely challenging environment for targeted therapeutic delivery, owing to its continuous contraction, dense vascularization, and dynamic extracellular milieu. The systemic administration of bioactive agents, including anti-inflammatory drugs, growth factors, and gene modulators, often leads to rapid clearance, nonspecific distribution, and unintended systemic effects such as immune activation, peripheral edema, and ectopic angiogenesis [170,171]. These limitations underscore the critical need for delivery strategies that ensure site-specific retention, prolonged bioactivity, and minimal off-target drug exposure.

Hydrogels have emerged as promising vehicles to meet these requirements, functioning as locally implantable, tunable reservoirs capable of encapsulating diverse therapeutic payloads while maintaining mechanical compatibility with beating cardiac tissues. Their high water content, customizable degradation profiles, and capacity for sustained or stimuli-responsive release render them ideal for myocardial applications. Recent advances have enabled hydrogel-based systems to deliver small molecules, recombinant proteins, and nucleic acids directly to the injured myocardium, demonstrating their efficacy in modulating inflammation, promoting angiogenesis, attenuating fibrosis, and enabling gene modulation or editing. Furthermore, innovations in polymer chemistry and delivery mechanisms now permit the spatiotemporal control of release kinetics, allowing for alignment with distinct phases of myocardial injury and repair [42].

### 7.1. Rationale for Localized Delivery in the Heart

Following MI or cardiac surgery, the heart undergoes a cascade of structural and biochemical remodeling characterized by localized inflammation, ECM degradation, fibrotic scar formation, and hypoxic stress. The peri-infarct zone, marked by intense immune infiltration and dynamic tissue turnover, represents a therapeutically accessible niche for interventions aimed at promoting angiogenesis, attenuating inflammation, and mitigating maladaptive remodeling [172]. However, the biomechanical and physiological characteristics of the myocardium, including continuous contraction, high vascular perfusion, and efficient lymphatic drainage, pose formidable barriers to drug retention and sustained therapeutic action [63].

The systemic administration of agents such as corticosteroids, antifibrotic compounds, and proangiogenic cytokines has shown limited success in cardiac applications, largely owing to rapid systemic clearance, poor myocardial accumulation, and off-target effects, including immunosuppression, hypertension, and ectopic tissue proliferation [173,174]. These limitations have driven the development of localized delivery platforms that enable the spatially restricted and temporally sustained therapeutic exposure of the injured myocardium.

Many hydrogel systems, particularly those capable of in situ gelation, offer geometrically conformal drug depots that support the localized, sustained release of bioactive agents, including small molecules, proteins, and nucleic acids, while preserving structural integrity and bioactivity [96,175]. Moreover, hydrogel degradation and release profiles can be finely tuned by manipulating their crosslinking density, polymer chemistry, and responsiveness to local physiological cues, such as pH shifts, enzymatic activity, and oxidative stress [176].

By confining therapeutic action to the site of injury, hydrogel-based delivery systems enable high local drug concentrations while minimizing systemic exposure and associated toxicity. This precision is particularly advantageous in cardiac applications, where spatially targeted treatment is essential for enhancing regenerative outcomes without triggering adverse effects [42].

### 7.2. Hydrogel Carriers for Small Molecules and Proteins

Hydrogels have demonstrated substantial promise as delivery platforms for small-molecule drugs and protein therapeutics in cardiac repair owing to their capacity to localize, protect, and release bioactive agents in a sustained and controlled manner. One illustrative example involves the delivery of dexamethasone using a thermosensitive hydrogel composed of PCL-HEMA-grafted PNIPAAm in a rat model of MI. This formulation exhibited a sol–gel transition at body temperature, enabling in situ gelation upon injection into the infarct border zone. Sustained dexamethasone release over 14 days significantly reduced macrophage infiltration (CD68+), preserved ventricular geometry, and improved cardiac function, as evidenced by increased EF and fractional shortening. These therapeutic effects were not observed with free drug administration, underscoring the importance of localized, prolonged delivery in modulating post-MI inflammation and remodeling [177].

The hydrogel-mediated delivery of proangiogenic growth factors, such as VEGF and bFGF, has also shown notable therapeutic benefits. These proteins promote endothelial cell recruitment, neovascularization, and myocardial salvage [178], but are limited by their rapid degradation and systemic clearance [179]. Natural-polymer-based injectable hydrogels, including gelatin and alginate matrices, have been used to encapsulate these growth factors, providing localized depots that release their cargo over 1–2 weeks. For instance, gelatin hydrogels crosslinked with microbial transglutaminase and loaded with VEGF or bFGF significantly enhanced capillary density and reduced infarct size in mouse MI models, with associated improvements in wall thickness and fractional shortening compared with bolus injection or saline control groups [180]. Similarly, alginate-based VEGF delivery improved myocardial perfusion and EF in preclinical models, accompanied by an increased vessel density and reduced fibrosis [181]. These results emphasize the dual role of hydrogels in stabilizing fragile protein therapeutics and supporting a permissive microenvironment for angiogenic repair.

In addition to single-factor strategies, hydrogel-based co-delivery systems have demonstrated synergistic effects on cardiac regeneration. For example, alginate hydrogels engineered for the sequential release of IGF-1 and hepatocyte growth factor (HGF) reduced cardiomyocyte apoptosis, minimized infarct size, and improved ventricular remodeling and function in rat MI models [182]. In a chronic porcine MI model, a ureido-pyrimidinone-based hydrogel co-loaded with IGF-1 and HGF further enhanced EF, capillary density, and the activation of endogenous progenitor cells, outcomes not observed with monotherapy or systemic delivery [183]. These findings highlight the potential of temporally programmed hydrogel systems to coordinate complex regenerative pathways in the body.

Stimuli-responsive hydrogels add another layer of specificity that enables context-dependent drug release in response to specific disease cues. MMP-responsive systems, for example, exploit the elevated MMP activity in the infarcted myocardium to trigger site-specific release. A collagen hydrogel crosslinked with MMP-2/9–cleavable peptides and loaded with a TIMP–bFGF fusion protein promoted angiogenesis and mitigated ventricular remodeling in a rat model of MI [184]. Another study developed a PEG-based conductive hydrogel incorporating MMP-degradable linkers and a prolyl hydroxylase domain inhibitor to stabilize hypoxia-inducible factor 1-alpha. This system enabled protease-sensitive release, preserved electrical conductivity, and improved cardiac function in infarcted mice [185].

In addition to drug delivery, hydrogels have been shown to modulate immune responses after myocardial injury. A thermosensitive chitosan-based hydrogel co-delivered with bone-marrow-derived MSCs reduced endothelial pyroptosis and the expression of pro-inflammatory cytokines (IL-1β, IL-6, TNF-α, IL-18, and caspase-1) and improved cardiac function in a rat MI model [186]. Another study employed a hybrid hydrogel composed of decellularized cardiac ECM and an immunomodulatory glycopeptide sialyl Lewis X, which promoted M2 macrophage polarization, enhanced neovascularization, and preserved cardiomyocyte viability in both rodent and porcine models, resulting in improved contractility [187].

Taken together, these studies highlight the versatility of hydrogel-based delivery systems for small molecules and proteins in cardiac repair. By enabling localized, sustained, and stimulus-responsive therapeutic release, hydrogels address the key limitations of systemic therapies by enhancing their efficacy while minimizing systemic toxicity and immune-related complications.

### 7.3. Nucleic Acid Delivery and Gene Modulation Strategies

Hydrogels are increasingly being developed as delivery platforms for nucleic-acid-based therapeutics, including plasmids, small interfering RNAs (siRNAs), microRNAs (miRNAs), and CRISPR/Cas9 components, to enable localized and sustained gene modulation in the injured myocardium. These approaches offer precise spatiotemporal control over gene expression, allowing for the reprogramming of pathological remodeling, attenuation of fibrosis, and promotion of angiogenesis while minimizing the systemic risks associated with conventional gene therapies [188,189].

A foundational example employed a thermosensitive amphiphilic polymer hydrogel to deliver plasmid DNA encoding VEGF. In a rat MI model, the intramyocardial injection of a VEGF-loaded Pluronic hydrogel resulted in sustained transgene expression for up to two weeks, enhanced capillary and arteriole densities, and improved cardiac function in the rat model. Notably, gene expression was confined to the infarct border zone, with no detectable expression in remote tissues, underscoring the spatial precision of hydrogel-mediated gene delivery [190].

Protease-sensitive hydrogels also enable stimuli-responsive siRNA release. In one study, siRNA targeting MMP-2 was encapsulated within a hyaluronic acid hydrogel crosslinked with MMP-cleavable peptides. This construct protected the siRNA from degradation and allowed for on-demand release in the infarct region, resulting in suppressed MMP-2 expression, reduced fibrosis, and the preservation of ventricular wall thickness compared to naked siRNA administration [191].

Hydrogels have also been used to deliver miRNAs, which regulate the post-transcriptional processes involved in inflammation and tissue regeneration. In a porcine MI model, a gelatin-based hydrogel embedded with mesoporous silica nanoparticles was used to deliver miR-21-5p in a pH-responsive manner. This strategy attenuated M1 macrophage polarization, promoted angiogenesis, and improved cardiomyocyte viability without inducing adverse fibrotic remodeling [123]. In parallel, collagen hydrogels encapsulating extracellular vesicles enriched with miR-199a-3p stimulated cardiomyocyte proliferation and enhanced systolic function in a rodent infarction model [192].

Although most CRISPR/Cas9-based gene-editing approaches have relied on viral vectors or ex vivo-modified cells, hydrogels are emerging as potential non-viral platforms for in situ delivery [7]. For instance, liposome–hydrogel nanoparticles successfully delivered Cas9–sgRNA ribonucleoproteins in vivo with preserved editing activity in tumor models, suggesting their feasibility for cardiac applications [193]. Although hydrogel-mediated CRISPR delivery has not yet been demonstrated in the heart, related studies have shown that the CRISPR-mediated deletion of TLR4 in mesenchymal stromal cells improved their reparative capacity post-MI [194]. These results support the potential of hydrogel-based in situ cardiac gene editing, although preclinical validation in a myocardial setting remains necessary.

Hybrid platforms that combine gene modulators with additional regenerative agents are also being investigated. For example, an alginate-derived hydrogel encapsulating exosomes enriched with miR-126 and miR-146a enhanced angiogenesis and reduced infarct size in a rat model of MI. This dual-delivery approach leveraged both the paracrine effects of exosomes and spatial localization conferred by the hydrogel scaffold to achieve synergistic therapeutic outcomes [195].

Collectively, these findings highlight the transformative potential of hydrogel-based systems in gene modulation strategies for cardiac repair. Representative examples are summarized in Table 6, which illustrates the diversity of hydrogel formulations, delivery targets, and preclinical outcomes across various cardiac models. By creating a localized, protective, and tunable microenvironment for nucleic acid therapeutics, hydrogels can enhance delivery precision, extend bioactivity, and reduce the systemic toxicity of therapeutic agents. As RNA-based drugs and gene-editing tools continue to progress toward clinical adoption, the integration of intelligent stimulus-responsive hydrogel carriers is expected to play a pivotal role in enabling safe and effective cardiac gene therapy [189].

## 8. Conclusions and Future Perspectives

Hydrogels have emerged as transformative biomaterials in the evolving landscape of cardiac surgery, offering multifaceted platforms for myocardial repair, tissue engineering, adhesion prevention, and localized therapeutic delivery. Their unique combination of tunable mechanical properties, high biocompatibility, and capacity to recapitulate the structure and function of the native ECM renders them particularly well suited for the complex and dynamic environment of the heart. As demonstrated across diverse preclinical models, hydrogel-based strategies can enhance post-infarction tissue regeneration, support the fabrication of bioengineered valves and myocardial patches, prevent postoperative adhesions, and enable the spatially confined release of small molecules, proteins, and nucleic acid therapeutics.

Despite this progress, several translational challenges must be addressed before these systems can be widely adopted in clinical practice. Many hydrogel formulations require further optimization to meet the mechanical durability and regulatory standards required for cardiovascular applications. Their long-term safety, immunogenicity, and structural integrity, particularly in high-stress anatomical regions such as the aortic outflow tract or ventricular wall, must be validated through rigorous testing in large animal models and early-phase human trials. Additionally, technical hurdles remain regarding scalable manufacturing, reproducible crosslinking chemistries, and compatibility with minimally invasive or catheter-based surgical delivery platforms.

Looking forward, several strategic directions are likely to accelerate the clinical translation of hydrogel technologies. Responsive hydrogels that release their therapeutic cargo in response to biochemical or biomechanical cues, such as enzyme activity, pH changes, or mechanical strain, can enable real-time synchronization with tissue remodeling processes, thereby enhancing therapeutic precision and efficacy. Hybrid constructs that integrate hydrogels with load-bearing polymers or native ECM components offer a promising route for achieving biomechanical robustness while maintaining bioactivity, particularly in valve and myocardial patch applications. Concurrently, advances in 3D bioprinting and personalized medicine will facilitate the creation of anatomically and functionally customized hydrogel constructs tailored to patient-specific needs with a high spatial resolution.

Importantly, the regulatory landscape for hydrogel-based devices and combination products must evolve in parallel with technological advancements. The hybrid nature of these materials, which span devices, biologics, and drugs, necessitates updated frameworks for safety assessments, efficacy evaluations, and manufacturing validation. Continued interdisciplinary collaboration among biomaterial scientists, engineers, surgeons, and regulatory authorities is critical for streamlining the translation process and ensuring the development of clinically viable solutions.

In summary, hydrogels represent a rapidly advancing and highly adaptable platform for addressing the longstanding challenges of cardiac surgery. Through precise material design, responsive functionalization, and rigorous translational validation, hydrogel technologies have the potential to redefine therapeutic paradigms, transforming how cardiovascular tissues are protected, repaired, and regenerated in the future.

## Figures and Tables

**Table 1:** Representative challenges in cardiac surgery and corresponding hydrogel-based strategies.

Challenge	Clinical Context	Hydrogel-Based Solution	Potential Benefits
Post-infarction tissue necrosis	Myocardial infarction, surgical trauma	Injectable hydrogels for structural support and ECM mimicry	Enhances tissue preservation, limits adverse remodeling
Fibrotic scar formation	Post-MI remodeling, post-surgical healing	Anti-fibrotic or MMP-responsive hydrogels	Suppresses myofibroblast activation and fibrosis
Pericardial adhesion	Postoperative complications, redo surgeries	Epicardial or pericardial hydrogel barriers	Reduces adhesion severity and improves surgical access
Inefficient therapeutic delivery	Systemic delivery of drugs or cells	Localized hydrogel depots with sustained release	Minimizes systemic toxicity and improves targeting
Host–implant integration failure	Synthetic patches, grafts, or valves	ECM-mimetic or biointeractive hydrogel scaffolds	Improves cellular infiltration and long-term biointegration

MI, myocardial infarction; ECM, extracellular matrix; MMP, matrix metalloproteinase.

**Table 2:** Summary of hydrogel types used in cardiac applications.

Hydrogel Type	Representative Materials	Key Features	Limitations	Example Applications
Natural	Collagen, Gelatin, Alginate, Fibrin	Biocompatible, ECM-mimetic, cell-adhesive	Rapid degradation, weak mechanics	Cell therapy, drug delivery, vascularization
Synthetic	PEG, PVA, PAAm	Tunable, reproducible, mechanically robust	Bioinert, requires functionalization	Patches, anti-adhesion barriers, scaffolds
Hybrid	PEG-Gelatin, Alginate-PEG, ECM-composites	Balanced bioactivity and stability	Complex synthesis	Injectable supports, 3D bioprinting
Smart/Bioresponsive	PNIPAAm, MMP-degradable, conductive gels	Stimuli-triggered release, adaptive function	Design complexity, scalability	Electromechanical repair, controlled release

PEG, polyethylene glycol; PVA, polyvinyl alcohol; PAAm, polyacrylamide; ECM, extracellular matrix; PNIPAAm, poly(N-isopropylacrylamide); MMP, matrix metalloproteinase.

**Table 3:** Hydrogel-based strategies for myocardial repair and their therapeutic roles.

Strategy Type	Example Materials	Key Therapeutic Roles
Cardiac patches	PEG–fibrinogen, gelatin–CNT	Mechanical reinforcement, VEGF delivery, electromechanical restoration
Stem cell encapsulation	Fibrin, GelMA, hydrolyzed gelatin	Enhanced cell retention and viability, prolonged paracrine signaling
Angiogenic hydrogels	Gelatin–bFGF, alginate–VEGF	Capillary formation, improved perfusion, reduced infarct size
Immunomodulatory hydrogels	Alginate–Annexin A1, NapFFY–SN50–IL10	Macrophage polarization, suppressed inflammation, promoted regeneration
Multifunctional composites	MXene–gelatin, GelMA–MSN–miR-21-5p	Angiogenesis + immune modulation + electrical coupling
Preclinical and clinical systems	Alginate (Algisyl-LVR), Ventri-Gel	Structural support, reverse remodeling, improved EF and functional class

PEG, polyethylene glycol; CNT, carbon nanotube; GelMA, gelatin methacryloyl; bFGF, basic fibroblast growth factor; VEGF, vascular endothelial growth factor; MSN, mesoporous silica nanoparticle; EF, ejection fraction.

**Table 4:** Selected hydrogel-based anti-adhesion systems under clinical or preclinical evaluation, categorized by composition, crosslinking mechanism, delivery strategy, and degradation time.

Product Name	Material Composition	Crosslinking Mechanism	Delivery Method	Degradation Time
Seprafilm	Hyaluronic acid + carboxymethylcellulose	Physical (hydration gel)	Sheet (manual placement)	≈7 days
CoSeal	PEG-based hydrogel	Chemical (crosslinking of PEG derivative)	Sprayable (dual syringe applicator)	≈30 days
Intercoat	Hyaluronic acid-based	Physical	Sprayable	≈7–10 days
SprayGel	PEG + blue dye	Chemical (photopolymerization)	Sprayable (with light source)	≈7 days
Hydrofit	Aldehyde-amino PEG hydrogel	Chemical (Schiff base formation)	Sprayable	~2 weeks

PEG, Polyethylene glycol.

**Table 5:** Representative hydrogel-based strategies in valve and patch engineering.

Strategy/Material	Application	Key Features
Photocrosslinkable hyaluronic acid–GelMA hydrogel	Valve scaffold (VICs)	Low α-SMA expression; aligned ECM deposition
PEG–dECM microparticle composite hydrogel	Valve scaffold (VICs + ECs)	Enhanced cell infiltration; re-endothelialization
Collagen–HA hydrogel under shear stress	Endothelial response modeling	Endothelial alignment; cytoskeletal remodeling
GelMA–PCL nanofiber-reinforced scaffold	Leaflet reinforcement	High modulus; shape recovery under cyclic loading
Electrospun PCL/PLLA tri-leaflet scaffold	Stented TEHV	Functional under pulsatile flow; frame integration
3D printed GelMA–alginate valve leaflets	Anatomical valve model	Region-specific stiffness gradient; physiological motion
Bioprinted alginate–gelatin myocardial patches	Functional myocardial patch	Synchronous beating; early angiogenesis and alignment

GelMA, gelatin methacryloyl; PEG, polyethylene glycol; dECM, decellularized extracellular matrix; HA, hyaluronic acid; PCL, polycaprolactone; PLLA, poly(L-lactic acid); VIC, valvular interstitial cell; EC, endothelial cell; TEHV, tissue-engineered heart valve; ECM, extracellular matrix.

**Table 6:** Summary of hydrogel-based strategies for nucleic acid delivery in cardiac repair.

Delivery Target	Hydrogel Type	Responsive Feature	Therapeutic Outcome
Plasmid DNA (VEGF)	Thermosensitive amphiphilic polymer (Pluronic)	Passive release	↑ Angiogenesis, ↑ EF
siRNA (MMP-2)	HA + MMP-cleavable peptide crosslinks	MMP-sensitive	↓ Fibrosis, ↑ wall thickness
miRNA (miR-21-5p)	Gelatin + silica nanoparticles	pH-sensitive	↓ M1 macrophages, ↑ viability
miRNA (miR-199a-3p EVs)	Collagen	Passive release	↑ Proliferation, ↑ EF
CRISPR–Cas9	Liposome–hydrogel nanoparticles	Not tested in heart	Preserved editing activity
Exosome + miR-126/146a	Alginate	Dual delivery	↑ Angiogenesis, ↓ infarct size

VEGF, vascular endothelial growth factor; siRNA, small interfering RNA; MMP, matrix metalloproteinase; miRNA, microRNA; HA, hyaluronic acid; EF, ejection fraction.

## Data Availability

No new data were created or analyzed in this study.

## Abbreviations

The following abbreviations are used in this manuscript:

3D	Three-dimensional
ECM	Extracellular matrix
PEG	Polyethylene glycol
PVA	Polyvinyl alcoho
TNF-α	Tumor necrosis factor-alpha
IL	Interleukin
TGF-β	Transforming growth factor-beta
MI	Myocardial infarction
VEGF	Vascular endothelial growth factor
IGF-1	Insulin-like growth factor 1
PNIPAAm	Poly(N-isopropylacrylamide)
MSC	mesenchymal stem cell
iPSC-CM	Induced pluripotent stem cell-derived cardiomyocyte
MMP	Matrix metalloproteinase
GelMA	Gelatin methacryloyl
PAAm	Polyacrylamide
EF	Ejection fraction
bFGF	Basic fibroblast growth factor
SMA	Smooth muscle actin
TLR	Toll-like receptor
NYHA	New York Heart Association
PACM	Poloxamer–alginate–calcium chloride
TEHV	Tissue-engineered heart valve
VIC	Valvular interstitial cell
PCL	Polycaprolactone
HGF	Hepatocyte growth factor
siRNA	Small interfering RNA
miRNA	MicroRNA
